# The challenges of magnetic resonance imaging and the promise of portable neurotechnology for advancing scientific discovery about neurodevelopmental disorders

**DOI:** 10.3389/fnrgo.2026.1884952

**Published:** 2026-09-23

**Authors:** Nancy Raitano Lee

**Affiliations:** Department of Psychological and Brain Sciences, Drexel University, Philadelphia, PA, United States

**Keywords:** electroencephalography (EEG), functional near infrared spectrocopy (fNIRS), magnetic resonance imaging (MRI), magnetoencephalography (MEG), neurodevelopmental disorders (NDD)

## Introduction

Neurodevelopmental disorders (NDDs) are conditions that impact cognition, behavior, social, and motor development and have their onset in the “developmental period” (American Psychiatric Association, 2013). This diverse group of conditions includes diagnoses characterized by a range of challenges—from focal limitations in one domain of functioning (e.g., Speech Sound Disorder) to global limitations in intellectual and adaptive functioning (e.g., Intellectual Disability). Given that NDDs are first evident in infancy and early childhood, developmental scientists have emphasized the importance of studying these conditions from the earliest point possible so that effective interventions can be developed to promote optimal outcomes (Masten and Cicchetti, 2010).

However, this scientific ideal is not without its challenges, as studying cognition, behavior, and neural functioning in infants and young children, particularly those with atypical cognition and behavior, requires special approaches to ensure that research findings are valid. This is particularly challenging when studying ***neural functioning***, as the demands of some neuroimaging approaches (e.g., magnetic resonance imaging) can exceed the developmental capabilities of the individuals who are the focus of the scientific investigation. This is a barrier to research for NDDs and likely impacts the field's ability to discover the mechanisms driving atypical brain and cognitive development.

In this Opinion piece, challenges using magnetic resonance imaging (MRI) and other neuroimaging tools embedded in healthcare settings (e.g., magnetoencephalography) with individuals with NDDs will be described and the dearth of functional MRI research in one group, children with Down syndrome, will be highlighted. Then recommendations for future research will be put forth, with an emphasis on the utility of portable neurotechnology. Finally, recommendations will be made for neuroimaging studies of NDDs.

## Neuroimaging and NDDs

As healthcare tools, neuroimaging approaches such as MRI, were developed within the medical model of illness, in which a healthcare provider directs care and patients are expected to comply with provider directives (Kumar and Chattu, 2018). In this model, the patient needs to adapt to the medical environment, rather than the medical environment adapting to the patient. Neuroimaging research that uses tools like MRI is also embedded in the medical model, and this presents barriers to research participation for groups with NDDs, as will be described next.

### Barriers

Participation in neuroimaging studies conducted in healthcare settings presents many barriers to individuals with NDDs even before the neuroimaging procedures are commenced. Examples include (a) traveling to the medical/research facility, (b) traversing complex, overstimulating environments, (c) waiting for care, and (d) communicating with care providers. These tasks and others have been cited as barriers to healthcare and research participation by individuals with NDDs and their families (Haas et al., 2016; Stein Duker et al., 2022). Once these steps are completed, individuals with NDDs and their families face unique challenges inherent to neuroimaging procedures, like MRI. For example, participants need to (a) lie still with little head motion, (b) tolerate being in an unfamiliar, confined space, and (c) cope with the sensations (noises; vibrations) associated with MRI machines (Greene et al., 2016). These challenges likely contribute to lower research participation for individuals with intellectual limitations, as described next.

### Evidence for reduced neuroimaging participation related to intellectual/developmental level

Among groups with NDDs, such as autism spectrum disorder, there is evidence that intellectual level impacts inclusion in neuroimaging research. For example, Jack and Pelphrey (2017) queried the National Database for Autism Research [NDAR, which is now housed under the larger National Institute of Mental Health Data Archive (NDA)] to identify the proportion of neuroimaging studies that focused on different subgroups of autistic individuals. They found that just over 10% of participants had verbal and performance IQs greater than 1 standard deviation below the population mean and just under 1% of these participants had any neuroimaging data. This is in spite of the fact that close to 40% of autistic children have intellectual disability [ID; (Shaw, 2025)] which is characterized by intellectual ability levels ~2 or more SDs below the population mean (American Psychiatric Association, 2013).

Consistent with these findings, research conducted on Down syndrome, the most common genetic cause of ID (Antonarakis, 2017), has documented lower success rates for MRI acquisition for those with lower IQs. Specifically, Lee et al. (2016) reported a trend for a lower average IQ among participants who could not complete MRI scanning procedures (IQ = 49) relative to those who could (IQ = 58). In addition, age was significantly associated with scanning success in Lee et al. (2016). Participants who were not able to provide usable MRI data were younger on average, suggesting that developmental level (as approximated by chronological age in this study) also relates to the ability to complete MRI procedures.

The challenges collecting useable MRI data from groups with NDDs are likely to be magnified when *functional* (as opposed to structural) MRI is considered. During structural MRI data acquisition, participants are able to watch a movie or sleep. In contrast, during functional MRI (fMRI) data acquisition, participants either need to be (a) awake but without external stimulation like a movie (i.e., for resting state functional MRI; rsfMRI) or (b) completing a task while MRI data are acquired (i.e., for task-based fMRI).

To query how MRI parameters (rsfMRI vs. task-based fMRI vs. other) relate to (a) the quantity of published studies and (b) the age of the participants, a Web of Science literature search was completed focused on Down syndrome (DS), as a case example. The following search terms were used: Title = “Down^*^ syndrome” and Abstract= MRI or “magnetic resonance imaging.” A total of 198 published articles were identified. Among these, 83 abstracts were deemed relevant in that they described quantitative neuroimaging investigations of infants, children, or adults (as opposed to studies focused on other organs or brain-focused case series, for example).

Among the 83 study abstracts reviewed, 14 included fMRI. (This excluded studies employing positron emission tomography or single-photon emission computed tomography only). Of the 14 studies that included fMRI specifically, 4 employed task-based fMRI. With regard to the age of participants, 58 studies included adults only; 12 included adult and child participants; and 13 included children (or children and infants) only. When the 14 fMRI investigations were examined, none included child participants exclusively (though one included mostly child participants). Thus, for DS, few fMRI (particularly task-based fMRI) studies exist, and most focus on adults. This is problematic for scientific discovery about the brain bases of the complex learning difficulties faced by children with DS and highlights the need for scientists to develop approaches to remove barriers associated with MRI, as discussed next.

### Removing barriers: the promise of portable neurotechnologies for participants with NDDs

In order to create developmentally informed, mechanistic theories of the neural bases of the cognitive underpinnings of NDDs, alternative neuroimaging approaches, particularly task-based functional neuroimaging approaches, are needed to permit greater participation. One approach to increase participation is to engage children and families in extensive behavioral training to help them gain the skills necessary to participate in neuroimaging studies within a healthcare setting. There are several examples of behavioral protocols that have been developed to improve research participation for these studies, including those for MRI (Nordahl et al., 2016) and magnetoencephalography (Kuschner et al., 2021).

Alternatively, the neuroimaging technology can be modified to meet the needs of the participant. Portable neurotechnologies, such as functional near infrared spectroscopy (fNIRS) and electroencephalography (EEG), are two approaches that have the potential to remove several barriers. Figure 1A presents a summary of some of the barriers neuroimaging approaches, like MRI, present for participants with NDDs. First, the barrier is presented. Then evidence for the relevance of the barrier to participants with NDDs is provided. Finally, a description of how portable neurotechnology may address these barriers is offered.

**Figure 1:**
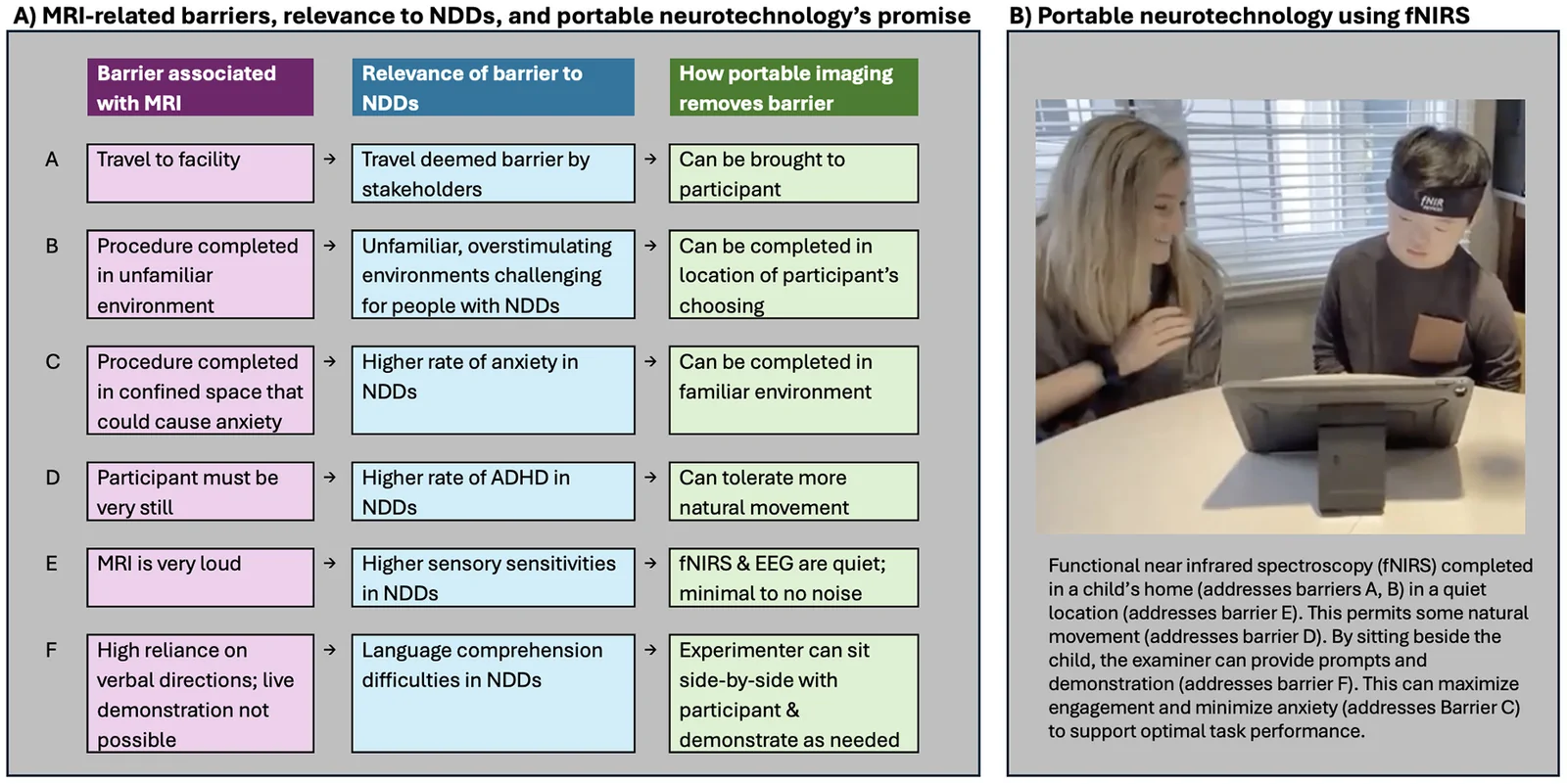
Barriers to magnetic resonance imaging for neurodevelopmental disorders and the promise of portable neuroimaging methods. **(A)** Illustrates barriers of magnetic resonance imaging (MRI), how these barriers impact individuals with neurodevelopmental disorders (NDDs) in particular, and how portable neurotechnologies such as functional near infrared spectroscopy (fNIRS) or electroencephalography (EEG) may help to reduce barriers. **(B)** Presents an example of how fNIRS can be used in a child's natural environment to reduce the impact of the barriers presented in panel **(A)**. EEG, Electroencephalography; fNIRS, functional near infrared spectroscopy; MRI, Magnetic Resonance Imaging; NDDs, Neurodevelopmental Disorders.

By definition, portable neurotechnologies, which can be brought into a participant's home, can eliminate the first two barriers to participation presented in Figure 1A—travel and completion of procedures in an unfamiliar environment, which can be especially stressful for families of individuals with autism spectrum disorder (Haas et al., 2016; Stein Duker et al., 2022). Portable neurotechnologies, such as fNIRS and EEG, also eliminate aspects of the MRI experience that can be especially problematic for individuals with NDDs, including being required to remain completely still in a confined space and tolerate loud noises. Elimination of these barriers is particularly crucial to increased participation, as individuals with NDDs present with higher rates of anxiety (Zhou et al., 2026) and attention-deficit/hyperactivity disorder (ADHD; (Perera et al., 2022)) and are more likely to experience sensory sensitivities (Tomchek and Dunn, 2007; Isralowitz et al., 2023) than neurotypical peers. Finally, portable neurotechnologies allow the examiner to interact with the participant in an ecologically valid manner. Examiners are able to sit next to or across from the participant and demonstrate how to do a task. This is crucial for easing anxiety, increasing engagement, and reducing the language demands of tasks by permitting demonstration and gestures. This latter component is crucial for people with NDDs who experience high rates of communication difficulties (Filipe et al., 2023) which can serve as barriers to functional MRI studies where the participants are separated from the examiner and must follow oral instructions over a speaker in the MRI scanner. Figure 1B illustrates how fNIRS studies can be completed with participants in their naturalistic environment, highlighting the removal of barriers associated with MRI and other neuroimaging tools embedded in healthcare settings.

## Discussion and recommendations for future research

In this Opinion piece, the barriers associated with MRI research for individuals with NDDs were presented alongside the promise of portable neurotechnologies to reduce barriers. The promise of these approaches must also come with an acknowledgment that no neuroimaging approach is appropriate for all participants. fNIRS, for example, requires participants to tolerate wearing a snug band or cap on their head during task completion; EEG requires participants to tolerate electrode placement and associated sensations. In order to advance research on the neural bases of the cognitive underpinnings of NDDs, there is a need to identify which neuroimaging approaches work well for which participants and which approaches do not. This is essential for the study of NDDs, as these conditions are associated with a great deal of heterogeneity (Beversdorf, 2024), making a one-size-fits-all approach problematic.

Transparency about the generalizability and limitations of neuroimaging research findings is of the utmost importance. Thus, the following suggestions are provided for researchers to consider when designing neuroimaging research studies focused on NDDs. First, researchers should consider embedding neuroimaging investigations into broader behavioral phenotyping studies. When advertising for these studies, it should be clear to families that the successful completion of neuroimaging procedures is not necessary for participation. Rather, it is one component of the study and there is no expectation that all participants will complete these procedures. The hope is that this will reduce the impact of family self-exclusion for neuroimaging studies (i.e., families who do not think their child could complete neuroimaging procedures choose not to sign up). Once participants are enrolled, it will be essential that all participants complete behavioral phenotyping procedures so that researchers can carefully characterize the phenotypes of participants who are and are not able to tolerate neuroimaging procedures. Although many studies are following guidelines regarding the documentation of the inclusion and exclusion of participants in neuroimaging research (e.g., Nichols et al., 2016), more extensive approaches must be taken to *characterize* individuals who are excluded (rather than just noting the number of participants who were excluded). Suggested characteristics to document include age, sex, global cognitive level, autistic traits, adaptive behavior, and maladaptive behavior.

The goal of such documentation is to increase transparency about the generalizability of the findings—i.e., to whom research findings apply and perhaps even more importantly, to whom they do not apply. This documentation may provide the necessary scientific justification for the development of alternative technologies that fit the needs of participants with NDDs for whom existing approaches are not appropriate. It is hoped that this will increase access to research, and in turn, will increase equity in accessing interventions for all individuals with NDDs.
